# Optimization of Hyaluronate-Based Liposomes to Augment the Oral Delivery and the Bioavailability of Berberine

**DOI:** 10.3390/ma14195759

**Published:** 2021-10-02

**Authors:** Hussam I. Kutbi, Hani Z. Asfour, Ahmed K. Kammoun, Alaa Sirwi, Simona Cavalu, Heba A. Gad

**Affiliations:** 1 Department of Pharmacy Practice, Faculty of Pharmacy, King Abdulaziz University, Jeddah 21589, Saudi Arabia; hkutbi1@kau.edu.sa; 2 Department of Medical Microbiology and Parasitology, Faculty of Medicine, Princess Al-Jawhara Center of Excellence in Research of Hereditary Disorders, King Abdulaziz University, Jeddah 21589, Saudi Arabia; hasfour@hotmail.com; 3 Department of Pharmaceutical Chemistry, Faculty of Pharmacy, King Abdulaziz University, Jeddah 21589, Saudi Arabia; akammoun@kau.edu.sa; 4 Department of Natural Products, Faculty of Pharmacy, King Abdulaziz University, Jeddah 21589, Saudi Arabia; asirwi@kau.edu.sa; 5 Faculty of Medicine and Pharmacy, University of Oradea, P-ta 1 Decembrie 10, 410087 Oradea, Romania; 6 Department of Pharmaceutics and Industrial Pharmacy, Faculty of Pharmacy, Ain Shams University, Cairo 11566, Egypt

**Keywords:** liposomes, hyaluronic acid, full-factorial, berberine, bioavailability

## Abstract

Various perspectives had been utilized to enhance the poor intestinal permeability and bioavailability of drugs with low water solubility. Berberine (Brb) is a unique molecule that possesses multiple therapeutic activities such as antimicrobial, anti-inflammatory, antioxidant and anti-hyperglycemic effects. To improve Brb permeability and bioavailability, this study presents a newly developed formulation, namely Brb hyaluronate-based liposomes, prepared by using film hydration method and characterized by dynamic light scattering measurements, entrapment efficiency percentage (EE%), transmission electron microscope (TEM), in vitro drug release and physical stability. The bioavailability of the selected formulations was assessed in vivo after oral administration to rats. The results revealed an enhanced effect of hyaluronic acid on the entrapment efficiency, reaching 78.1 ± 0.1% with mean size 520.7 ± 19.9 nm. Sustained release of Brb was recorded up to 24 h in comparison to Brb solution. Physical stability was maintained for three months at refrigeration temperature. Results of pharmacokinetics studies indicated the potential of the liposomal formulation to increase the oral bioavailability of Brb and to accelerate its entry into the bloodstream. The obtained results are accredited to the lipophilic nature of the prepared system, resembling the structural features of bio-membrane, in addition to their small size that enhances intestinal penetration.

## 1. Introduction

There is an increasing interest in the utilization of naturally existing compounds with potential pharmacological effect in the management of different diseases. Berberine is a wonderful bioactive alkaloid isolated from some medicinal Chinese herbs [1]. Brb are widely applied in Chinese medicine for the management of hypertension and inflammatory conditions [2]. The valuable properties of Brb is attributed to its diverse pharmacological actions including its antimicrobial, antiprotozoal, antidiarrheal, anti-inflammatory, antioxidant [3], anticancer [4], antiviral [5], antidiabetic effects [6], in addition to its beneficial effect on the cardiovascular and central nervous systems [7,8].

All these activities could permit the use of Brb in the treatment of many diseases. However, Brb properties have limited its wide clinical applications. Brb is characterized by low aqueous solubility (1 mg/mL) and a partition coefficient of log *p*-value = −1.5. This makes it a class III drug in the biopharmaceutical classification system, indicating its poor membrane permeability that resulted in poor gastrointestinal absorption and low bioavailability [9]. The low aqueous solubility of Brb is attributed to its self-aggregation upon contact with GIT fluid. Moreover to Brb low solubility, the low bioavailability is assigned to the effect of drug efflux pump, which acts as an obstacle for the entrance of many drugs [10], the first hepatic pass effect and the rapid metabolism by CYP 450-dependent processes [2,11]. It has been reported that Brb has an efflux ratio of 13.8 that means that Brb is a good substrate for P-glycoprotein [12]. Encapsulation of active ingredients into lipid-based nanocarriers was a golden solution for the problems of low solubility and permeability that could enhance both drug penetration and bioavailability. Lipid-based nanocarriers could enhance intestinal absorption through different pathways including uptake through microfold cells (M-cell), transcellular and paracellular permeation pathways [13].

Liposomes are one of the most widely utilized lipid based nanocarriers for enhancing drugs bioavailability via the oral route due to their biocompatibility, safety and versatility [14]. Many studies have been carried to increase stability permeability and biodistribution of liposomes and enhance drug encapsulation [15,16,17]. Trials include coating liposomes using different polymers. Li et al. formulated silica coated flexible liposomes to increase liposomes structural integrity against the harshened environment of the GIT [18]. Other trials involved altering the composition of the liposomes (either lipid bilayer or core). Catalan-Latorre et al. formulated eudragit and hyaluronan liposomes to ameliorate the gastro-enteric the bioavailability and stability of curcumin [19]. Hathout et al. formulated gelatinized core liposomes to decrease hydrophilic drug leakage and to increase liposomes stability [20].

Based on the above findings, the goal of the current study was to formulate a novel formulation, namely hyaluronate-based liposomes entrapping berberine using film hydration method and to evaluate the effect of different preparation parameters on the physicochemical properties of the prepared vesicles. Optimization of the formulation variables was performed using full factorial design. Moreover, the potential of the investigated system to enhance the lipophilicity and bioavailability of Brb and to sustain its in-vivo release rate after oral administration to rats were assessed.

## 2. Materials and Methods

Berberine (Brb) (CAS 633-65-8) (purity > 95%) was obtained from Nanjing Zelang Medical Technology Co. Ltd., Nanjing, China. Hyaluronic acid (HA) (CAS: 9004-61-9) was purchased from Acros Organics, Fisher Scientific, England, UK. Soya bean phosphatidylcholine (PDC) CAS 8002-43-5 and cholesterol (CH) (CAS 57-88-5) were purchased from Sigma-Aldrich (Darmstadt, Germany). Organic solvent including methanol and chloroform were obtained from El-Nasr Pharmaceutical Co. (Cairo, Egypt).

### 2.1. Preparation and Optimization of the Berberine Loaded Liposomes

Brb loaded liposomes were formulated adapting the thin-film hydration method [21]. Full-factorial design was implemented to evaluate the impact of different variables on the properties of the prepared vesicles. The investigated factors were the total lipid amount, Brb and HA amounts all at 2 levels as follows: 100 and 200 mg, 10 and 50 mg and 0 and 30 mg for the total lipid, Brb and HA respectively. The optimized responses were the entrapment efficiency percentage (EE%) and the mean size (MS) (Table 1). Briefly, organic solvent mixture of chloroform and methanol in a ratio of 2:1 was used to dissolve the lipid mixture of PDC and CH in a ratio of 7:3. Removal of the organic solvent to form a thin film was performed using a rotary evaporator flask (IKA Laboratories, Staufen, Germany) at 40 °C. Portion-wise addition of warmed HA solution (50 °C) was used to hydrate the formed film. The formed liposomal suspension was subjected to shaking for 1 h in a water bath (Kötterman, Uetze, Germany) maintained at 50 °C. 

### 2.2. Characterization of the Prepared Berberine Loaded Liposomes

#### 2.2.1. Determination of the Particle Size of the Prepared Liposomes

The particle size (PS) of Brb loaded liposomal formulations was measured at room temperature using Malvern Zetasizer Nano Series (Malvern Instruments, Malvern, UK) employing Dynamic Light Scattering [22]. Dispersions were four folds diluted with de-ionized water to avoid multi-scattering phenomena.

#### 2.2.2. Measurement of the Entrapment Efficiency Percentages (EE%) of Berberine Hydrochloride

An aliquot of the prepared Brb loaded liposomes was centrifuged at 25 °C (Hermle Labortechnik, Wehingen, Germany) at 15,000 rpm for 1 h to separate the nanoparticles [23]. The free un-entrapped Brb in the clear supernatant was measured at 340 nm using a UV—visible double beam Spectrophotometer (Shimadzu, Kyoto, Japan). 

The entrapment efficiency was determined as follows:

EE% = [(Wt − Wf)/Wt] × 100

where Wt represents the total drug concentration added to the formulation, Wf represents the amount of the free drug measured in the supernatant.

#### 2.2.3. Transmission Electron Microscopy (TEM) of the Prepared Berberine Loaded Liposomes

Morphological examination of the selected Brb hyaluronate-based liposomes (F4) was examined using TEM at 25 °C (Jeol Electron Microscope, Tokyo, Japan). An unstained droplet of the liposomes formulation was added to a carbon film-covered copper grid and left for three minutes. Then, a filter paper was used to remove the excess liquid and the sample was dried. Observation of the prepared sample by the microscope was performed at resolution of 20,000 KV.

#### 2.2.4. In Vitro Release Study of Berberine from the Prepared Liposomes

The in vitro drug release was performed using dissolution apparatus in phosphate buffer saline (pH 6.8) to simulate the physiological environment following oral administration [2,24]. The selected formulae (F3, F4) and Brb solution were exposed to the dissolution vessels containing 900 mL phosphate buffer saline of maintained at 37 ± 0.5 °C with a rotation of 100 rpm. At pre-determined intervals (15, 30, 45, 60, 90, and 120 min), aliquots of the medium were withdrawn, filtered with 0.45 µm filter and suitably diluted. The obtained samples were observed spectrophotometrically at a maximum wavelength of 340 nm to determine the concentration of the released Brb. All tests were conducted in triplicate.

#### 2.2.5. Assessing the Physical Stability of Berberine Hyaluronate-Based Liposomes

The mean size and entrapment efficiency of the selected berberine hyaluronate based liposomes (F4) were re-measured after three months storage at 2–8 °C (refrigeration) using the same procedures mentioned above. 

### 2.3. Pharmacokinetics Study

#### 2.3.1. Experimental Design

Animals were obtained from the animal house of the Faculty of Pharmacy, King Abdulaziz University, Jeddah, Saudi Arabia. The in vivo study protocol was approved by the Animal Ethics Committee of the Faculty of Pharmacy, King Abdulaziz University, Jeddah, Saudi Arabia, in adherence with the Declaration of Helsinki, the Guiding Principle in Care and Use of Animals (DHEW production NIH 80-23) and the Standards of Laboratory Animal Care (NIH distribution #85-23, reconsidered in 1985). The study included three randomly divided groups of male Wistar rats (N = 6/group): group 1, 2 and 3 received Brb solution, Brb liposomes (F3), Brb hyaluronate-based liposomes (F4) respectively. Rats were fasted for 16 h before the experiment and had free access to water. All groups administered Brb dose of 50 mg/kg body weigh via intra-gastric gavage. At different time intervals, blood samples (200 µL) were obtained from the medial canthus of the eye using capillary tubes after drug administration. Blood samples were centrifuged at 3000 rpm for 10 min at 4 °C to separate plasma [25].

#### 2.3.2. Sample Preparation and Calibration Curve

The analysis and the sample extraction technique were done applying the procedure adapted by Xue et al. 2013 [26] with certain modifications. In brief, one hundred μL plasma was transferred to test tube, then one hundred μL valsartan, 100 ng/μL as internal standard was added followed by 250 μL acetonitrile. Vortex the mixture for 1 min, followed by centrifugation for 15 min at 5300 rpm. One hundred μL of the supernatant was transferred to the total recovery vial. The injection volume was five μL. The calibration curve for Brb was assessed using blank plasma as a matrix. Stock solution of concentration 1 mg/mL of Brb was prepared using ethanol as solvent. From the stock solution a series of working solutions of Brb were prepared applying serial dilution technique. The calibration solutions were prepared by spiking separately blank plasma with Brb solutions. The calibration range was from 1.0 to 100.0 ng/mL of Brb with a fixed concentration of internal standard. All solutions were extracted and analyzed applying the developed method.

#### 2.3.3. Chromatographic System and Instrumentation

The analysis was done using triple quad mass spectrometer (Agilent 6460, Agilent Technologies, Santa Clara, CA, USA). The instrument coupled with electrospray ionization mass spec-trometer system. The system designed with a quaternary pump, and a column compartment (Palo Alto, Santa Clara, CA, USA and controlled by MassHunter software (version B.03.01, Build 3.1.346.0). The conditions of mass spectrometric measurements were a gas temperature, 330 °C; gas flow rate, 11 L/min; nebulizer pressure; 35 psi, and capillary voltage, 4300 V. The mass spectrometric settings were optimized for berberine, including the fragmentor voltage, dwell time, and collision energy voltage. The column used for separation at positive ion mode was anAgilent Eclipse Plus C8, 5 μm, 4.6 × 100 mm column. 

The mobile system composition was of acetonitrile: water containing 0.1% w/v formic acid in ratio 30: 70 v/v, with flow rate 0.5 mL/min. The TQ /MS conditions were optimized by applying different and alternative values of fragmentor voltage, dwell time, and collision energy (eV) and the optimum conditions were determined.

### 2.4. Data Analysis

Design Expert^®^ v. 7.0 (Stat Ease, Minneapolis, MN, USA) was used to obtain the models for the entrapment efficiency percentage (%) and the mean size. Analysis of the obtained results were performed using ANOVA at *p* < 0.05 using GraphPad Prism^®^ v.5.0 (GraphPad Software, La Jolla, CA, USA). Pharmacokinetics parameters including maximum concentration (C_max_) and time of maximum concentration (T_max_) were determined directly from the plasma concentration–time profiles. The area under the concentration–time curve (AUC) from time zero to test time (AUC_0−t_) was calculated using GraphPad Prism^®^ v.5.0 (GraphPad Software, La Jolla, CA, USA).

## 3. Results and Discussion

### 3.1. Optimization of the Berberine Loaded Liposomal Formulations

Brb loaded liposomes were successfully prepared using film hydration method that was easy to perform on small scale with the feasibility of scale up. In addition, thin film hydration method includes the hydration of the formed lipid film using preheated aqueous solution that enables decreasing the viscosity of HA. Optimization of the formulation parameters were achieved using Design Expert^®^ v. 7.0. Table 1 shows the optimized responses (EE% and PS), while Table 2 shows the obtained models and the results of ANOVA analysis for the responses of Brb liposomes with or without HA. The obtained models were significant showing a good fitting (r-squared and adjusted r-squared values above 0.98, with high predicted r-squared values). The difference between the predicted and the adjusted values was <0.2 indicating their high agreement. In addition, the adequate precision values of the models that reflect the signal-to noise ratio were high and above the value of four showing the sensitivity and the adequacy of the models [27].

**Figure 1:**
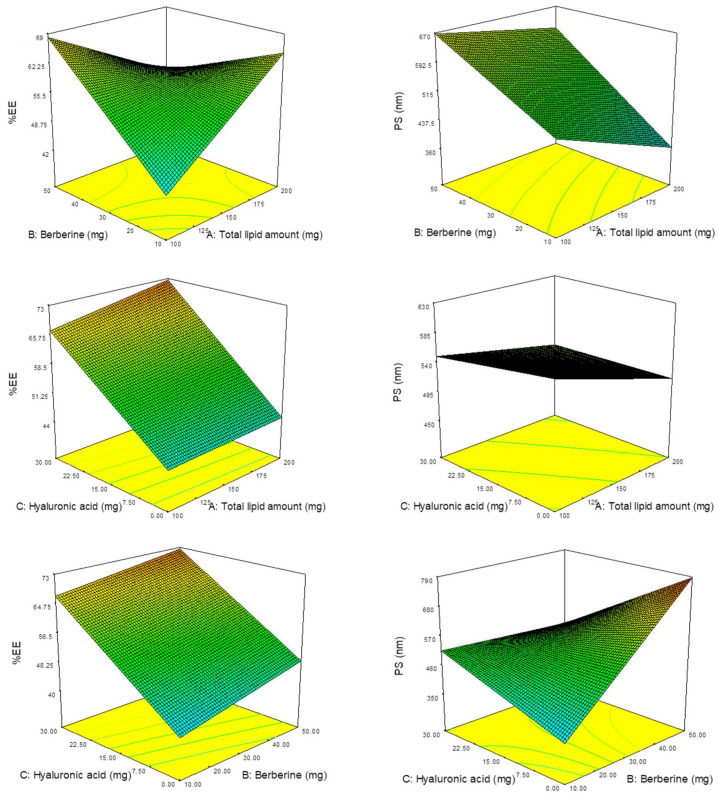
Three-dimensional (3D) response surface plots showing the effect of the independent variables on liposomes particle size and entrapment efficiency percent: A—total lipid amount (mg), B—berberine (mg), C—hyaluronic acid (mg).

Figure 1(A) shows the 3D surface plots of EE% as a model response. The plots show the effect of factors interaction on the Brb entrapment efficiency. The results indicate the effect of the different independent variables on the EE%, as investigated by the broad range of EE% from 29.91% to 78.12%. As revealed from the magnitude and sign of the estimated coefficients of the obtained equation, the presence of HA (C) has the significant effect on EE%. In addition the higher positive coefficient value observed for HA amount (C) in comparison to lipid and Brb amounts (A and B) indicates its more enhanced effect. 

The significant increase in the Brb EE% due to the presence of HA could be attributed to two main factors, firstly the ionic interaction between the quaternary ammonium group of Brb and the negatively charged carboxyl group of HA resulted in encapsulating more drug in the hyaluronated core of the prepared liposomes decreasing the drug leakage [28]. Secondly, the effect of HA presence on raising the viscosity of the inner core of the vesicles and the aqueous environment surrounding the lipid bi-layers with a subsequent decrease in the drug leakage from the vesicles. 

Figure 1(B) shows the 3D surface plots related to the particle size response modeling. The plots show the effect of factors interaction on the particle size. The results indicate the enhanced effect of the different independent variables on the PS, (ranging from to 389.7 to 825.7 nm). Upon inspection of the obtained equation, it can be deduced from the magnitude and sign of the estimated coefficients that Brb amount (B) has the major effect on PS. In addition the high positive coefficient value observed for Brb amount (B) indicates its significant effect. In contrast, the negative coefficient values observed for lipid amount (A) and HA present (C), indicating that these variables are insignificant in predicting PS. 

Consequently, the formulation prepared using 200 mg total lipid, 30 mg HA and 10 mg Brb (F4) with EE% of 78.12 ± 0.13% and PS equal to 520.7 ± 19.98 was selected for the further studies.

### 3.2. TEM

**Figure 2:**
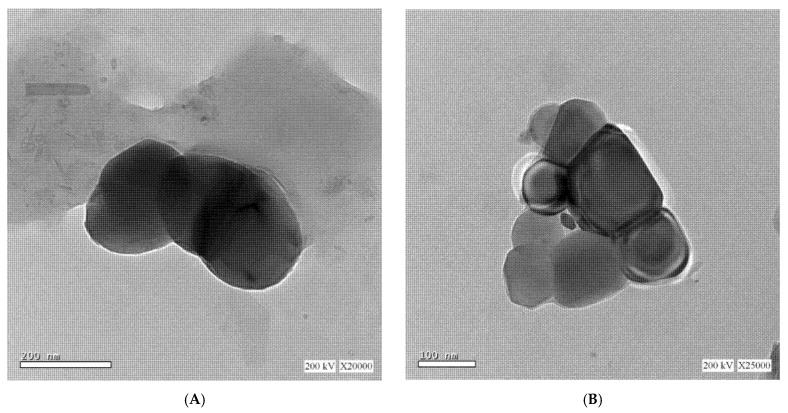
Transmission electron microscope of berberine hyaluronate based liposomes, showing the nearly spherical shape (**A**) and the multi-lamellar structure of the vesicles bilayer (**B**) of the prepared liposomes.

Figure 2 reveals the TEM images of selected Brb hyaluronate-based liposomes at room temperature. The figure reveals the multi-lamellar structure of the vesicles’ bilayer and the obvious deformation of the prepared particles. The dark color of the interior core of the vesicles indicates the occupation of the core with HA. The irregularity of the formed vesicles is assigned to the high viscosity of HA inside the vesicles at 25 °C resulting in vesicles deformation.

### 3.3. In Vitro Release Studies of Berberine from the Prepared Liposomes

**Figure 3:**
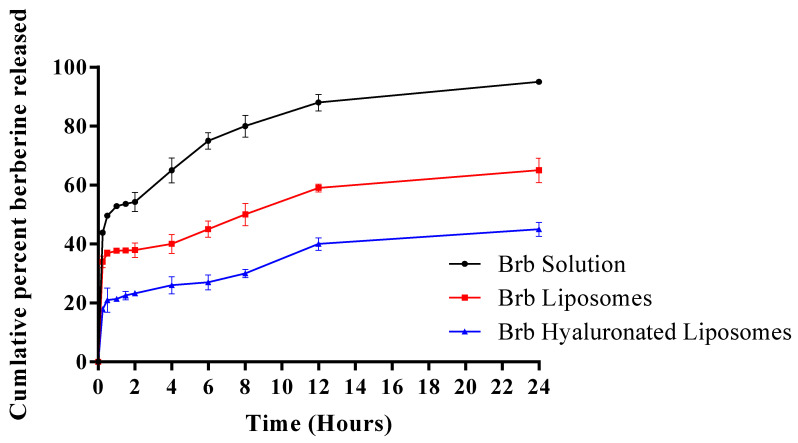
Cumulative in vitro release of berberine from different berberine formulations in phosphate buffer saline (pH 6.8), each result is the mean of three determinations ± SD.

It has been reported that Brb water solubility is affected by temperature condition and buffer constitution. Brb solubility in phosphate buffer was reported to be the highest among other buffers, like borate or phthalate buffer [9], therefore phosphate buffer was used to study Brb dissolution rate from the prepared vesicles to justify its sustained behavior. Figure 3 shows the in vitro release of Brb from the selected Brb hyaluronate-based liposomes (F4) in comparison to formulation containing the same lipid and drug amounts but without HA (F3) and Brb solution in phosphate buffer saline. As revealed Brb free drug solution showed fast release rate with 100% released within 6 h. Conversely, Brb liposomes showed a biphasic slower release rates especially hyaluronate-based liposomes; the initial burst effect may be attributed to the drug near the liposomes surface. F4 demonstrated the lowest initial burst effect as compared to F3 and drug solution; in addition, it revealed the slowest and the most sustained release profile. The enhanced sustained release of Brb from hyaluronate based liposomes may be assigned to the ionic interaction between the quaternary ammonium group of Brb and the negatively charged carboxyl ion of HA. In addition, HA is a hydrophilic water soluble polymer that was solubilized in the aqueous core of the formed vesicles and its presence could increase the viscosity of the inner core due to its high molecular weight and hence decreases the entrapped drug diffusion and improves its release control. These two factors contribute to the sustained release of berberine from Brb loaded HA based liposomes. The release of Brb from HA based liposomes showed Higuchi kinetics model after fitting the release data to different models based on the highest regression coefficients. Brb is well known for its low water solubility (1 mg/mL) and log P of –1.5, which implies its ability to be incorporated within the vesicles’ bilayer to a certain limit. In the present work berberine was used as its hydrochloride salt; therefore it was entrapped in the aqueous core of the liposomes. In addition, the presence of HA augment the incorporation of more drug in the hydrophilic core of the vesicles which is assured by the higher drug entrapment efficiency of hyaluronate-based liposomes. Our results are in accordance with a previous study [28,29].

### 3.4. Physical Stability

Results of the stability of F4 revealed acceptable stability with no statistically significant difference (*p* < 0.05) compared to initial values recording 502.57 ± 8.42 for the mean size and 77.74 ± 0.14% for the EE% after storage for 3 months. The results indicate the effect of the HA on supporting the physical stability of the prepared liposomes.

### 3.5. Pharmacokinetics Study

The parameters of HPLC method were validated according to ICH guidelines. The peak area ratios of Brb to internal standard were found linear with regression coefficient 0.9984 in the concentration range, 1.0 to 100.0 ng/mL of Brb. The concentrations of the Brb after extraction at different set points were calculated, referring to the obtained regression equation. The QQQ-MS optimum conditions were optimized where t_R_ showed 9.11 min, +MRM transition (*m*/*z*) ranged from 336.3 to 321.1, Dwell time was 200 ms at fragmentation voltage of 135 V and collision energy of 20 eV.

**Figure 4:**
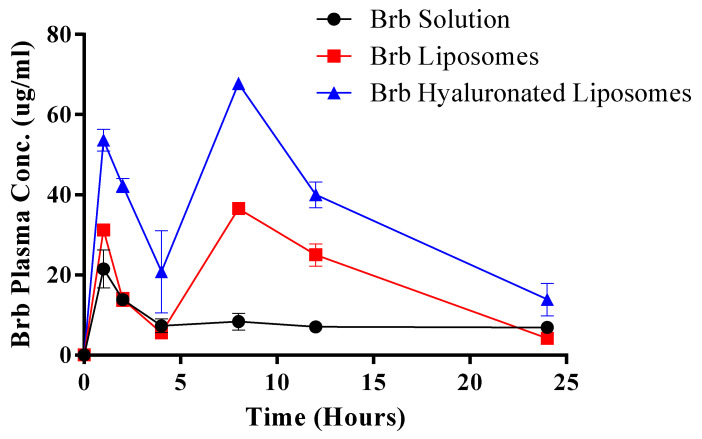
Plasma concentration profiles of berberine in rats after oral administrations of 50 mg/kg of berberine in various formulations. Each point represents mean ± standard deviation (N = 6).

Figure 4 shows a representative mass spectrum of berberine of concentration 1 ng/mL after extraction from plasma applying multiple reaction monitoring mode (MRM) to increase the sensitivity and selectivity of the measurement. The MRM transition mode applied for berberine was a precursor ion of *m/z* coupled with main fragment ion of berberine of *m/z* 321.1. The comparative pharmacokinetic profiles of Brb solution, Brb liposomes and Brb hyaluronate-based liposomes after oral administration to rats at a dose of 50 mg/kg are shown in Figure 4, and pharmacokinetic parameters (C_max_, T_max_, and AUC_0–24_) are summarized in Table 3.

It was obvious from Brb plasma concentration-time profile that Brb shows two absorption peaks, which may be attributed to enterohepatic circulation, which is in accordance with previous study [30]. However, other reasons may include the fast absorption of unentrapped adsorbed drug on the liposomes surface followed by the slower absorption through the lymphatic pathway [31].

C_max_ value increased in the order of Brb hyaluronate-based liposomes > Brb liposomes > Brb solution. The obtained C_max_ after oral administration of Brb solution was 21.5 ± 4.71 ng/mL, while C_max_ of Brb liposomes and Brb hyaluronate-based liposomes were 31.26 ± 0.109 and 53.61 ± respectively. Furthermore, the AUC_0–24_ of Brb solution, Brb liposomes and Brb hyaluronate-based liposomes were 194.8, 439.9 and 854 ng h/mL respectively. The higher C_max_ peaks and the greater AUC_0–24_ values of Brb liposomes and Brb hyaluronate-based liposomes indicate the greater absorption rate of Brb from liposomes. In addition, the delayed T_max_ (8 h) of Brb liposomes and Brb hyaluronate-based liposomes in comparison to Brb solution (1 h) indicates the slow sustained release of Brb, which correlates with the in vitro dissolution study. 

In addition, approximately 2.26- and 4.38-fold increases in relative bioavailability were achieved within Brb liposomes and Brb hyaluronate-based liposomes, respectively, when compared with the free Brb solution. Results of pharmacokinetics studies indicate that the oral bioavailability of Brb was increased and the entrance of Brb into the bloodstream was accelerated upon its encapsulation into vesicular system. The obtained results are accredited to the lipophilic nature of the prepared system, resembling the bio-membrane, in addition to the vesicles small size that enhances intestinal penetration [32]. 

## 4. Conclusions

The present study suggests that the hyaluronate-based liposomes are suitable formulations for successful delivery of Brb. Different formulation variables (lipid, drug and hyaluronic acid amounts) have a significant effect on the physicochemical characteristics of the prepared system using film hydration method. The physicochemical characterization and the results of in vitro release displayed multi-lamellar vesicles with acceptable particle size, high Brb entrapment efficiency and sustained drug release for 24 h. The presence of hyaluronic acid as a main component in liposomes preparation was able to slow berberine diffusion from the vesicles. Moreover, observing the pharmacokinetic behavior after oral administration of Brb hyaluronate-based liposomes to rats could improve lipophilicity and bioavailability of the investigated system compared to Brb solution and Brb liposomes prepared without hyaluronic acid. Further investigations are required in order to obtain smaller liposomal formulations, with multiple applications, such as targeted and controlled delivery of the bioactive compound, in addition to pharmacodynamics studies based on the diseases.

## Figures and Tables

**Table 1:** Composition, mean size and % EE of various berberine loaded liposomes.

Formulation	Variables (Independent Factors)		Responses (Dependent Variables)	
	Berberine:Lipid Ratio	Sodium Hyaluronate (mg)	MS (nm) ± SD	EE% ± SD
F1	1:10	0	494.9 ± 2.6	29.9 ± 0.6
F2	1:10	30	514.3 ± 9.7	55.9 ± 0.4
F3	1:20	0	207.1 ± 8.5	51.2 ± 0.5
F4	1:20	30	520.7 ± 19.9	78.1 ± 0.1
F5	1:2	0	748.5 ± 27.5	58.5 ± 0.2
F6	1:2	30	586.7 ± 36.6	77.9 ± 0.1
F7	1:4	0	825.7 ± 6.1	39.0 ± 0.1
F8	1:4	30	389.7 ± 46.8	67.0 ± 0.3

Total lipid: phosphatidylcholine and cholesterol in a ratio of 7:3. MS: mean size, % EE: percent entrapment efficiency. Each result is the mean of three determinations ± SD.

**Table 2:** Results of ANOVA analysis of entrapement efficiency and mean size of the berberine loaded liposomes.

EE (%)		MS
Suggested Model	Factorial	Factorial
Equation	% EE = +57.22 + 1.66 × A + 3.41 × B + 12.53 × C − 9.24 × A × B + 1.19 × A × C − 0.68 × B × C + 0.98 × A × B × C	PS (nm) = +535.94 − 50.17 × A + 101.68 × B − 33.10 × C + 20.21 × A × B + 2.51 × A × C − 116.36 × B × C − 71.05 × A × B × C
r^2^	0.9996	0.9877
Adjusted r^2^	0.9995	0.9824
Predicted r^2^	0.9992	0.9724
Adequate precision	220.858	43.267

**Table 3:** The calculated pharmacokinetic parameters of rats in different groups after administration of a single oral dose of berberine of 50 mg/kg body weight.

	Brb Solution	Brb Liposomes	Brb Hyaluronate Based Liposomes
C_max_ (ng/mL)	21.51 ± 4.71	36.54 ± 0.77	67.79 ± 1.12
T_max_ (h)	1	8	8
AUC	194.8 ± 6.73	439.9 ± 14.39	854 ± 23.69
Relative bioavailability	-	2.26	4.38

Data represents mean ± standard deviation.

## Data Availability

All data are reported in the manuscript.

## Abbreviations

Brb	berberine
HA	hylauronic acid
TEM	transmission electron microscope
PDC	Soya bean phosphatidylcholine
CH	cholesterol
MS	mean size
PS	particle ssize
EE	entrapment effificency
